# A Unique Presentation of Solid Pseudopapillary Neoplasm of the Pancreas Requiring Pancreaticoduodenectomy Without Pancreatojejunostomy: A Case Report and Literature Review

**DOI:** 10.7759/cureus.63603

**Published:** 2024-07-01

**Authors:** Alexis L Carmona, Sameh A Fayek

**Affiliations:** 1 Medicine, University of California (UC) Riverside School of Medicine, Riverside, USA; 2 Surgery, University of North Texas Health Science Center, Fort Worth, USA; 3 Surgery, Faculty of Medicine, Cairo, EGY

**Keywords:** pancreatobiliary malignancy, gastrojejunostomy, pancreas, pancreaticoduodenectomy, solid psuedopapillary neoplasm

## Abstract

Solid pseudopapillary neoplasms (SPNs) of the pancreas are rare tumors that predominantly affect young females and are typically located in the body and tail of the pancreas. Here, we present the case of a 59-year-old male with a large, heavily calcified SPN in the pancreatic head. His surgical history includes an aborted pancreaticoduodenectomy due to vascular involvement, followed by a gastrojejunostomy. Twenty years after the initial discovery, a pancreaticoduodenectomy was performed - the first of its kind - where the pancreas was completely atrophied, and no pancreaticojejunostomy was performed. Histological examination revealed typical features of SPN. This case demonstrates that even with relatively large lesions in a male patient over an extended duration, SPNs can still exhibit favorable features, highlighting the absence of specific preoperative markers for aggressive tumors. Therefore, unless there is an absolute contraindication, complete resection of all SPNs remains advisable.

## Introduction

Solid pseudopapillary neoplasms (SPNs) of the pancreas are among the least prevalent tumors, accounting for approximately 2% of all pancreatic neoplasms, and they are often misdiagnosed [1]. Classically, SPNs are large, encapsulated masses composed primarily of solid components with a mixture of cystic and hemorrhagic elements; however, occasionally, the dominant feature is the cystic component, and thus, the lesion is considered within the diverse group of pancreatic cysts [1]. Recently, pancreatic cysts have been more frequently identified with increased use and improved sensitivity of imaging techniques [2]. Pancreatic cysts can be the presenting feature of a heterogeneous group of diseases and are generally divided into simple retention cysts, pseudocysts, and cystic neoplasms, including SPNs, serous cystadenomas, mucinous cystadenomas, and intraductal papillary mucinous neoplasms [2]. Appendix A summarizes the key differentiating factors for each type of pancreatic neoplasm [3]. These carry different biological behaviors; thus, an accurate diagnosis is essential to guide management. Endoscopic ultrasound (EUS) and fine-needle aspiration (FNA) with samples analyzed for cytology, chemistry, and oncogenes became a cornerstone in resolving such a dilemma [4].

SPNs are predominantly reported in patients under 25 years with a female-to-male ratio of 9.5:1 and commonly within the body and tail of the pancreas [2,5]. Surgical resection is indicated once an SPN is identified, and the procedure type is determined based on the tumor’s location [6]. Surgery is often curative, and tumor recurrence is considered low [7].

Here, we report a unique case of an SPN presenting in an older male in the head of the pancreas. Contrary to the literature, despite the patient’s gender, the large size of the tumor, and two decades of course, the tumor histologic features were favorable, and the patient is considered cured with pancreaticoduodenectomy.

## Case presentation

A 37-year-old male patient with known high cholesterol and chronic alcohol intake was initially diagnosed with a pancreatic cyst that was incidentally discovered during the workup for a brain arteriovenous malformation. A pancreaticoduodenectomy was attempted four years later; however, this was aborted as the tumor was described to be vascularized and adherent to the superior mesenteric and portal vein, and the procedure was abbreviated to just a gastrojejunostomy.

**Figure 1:**
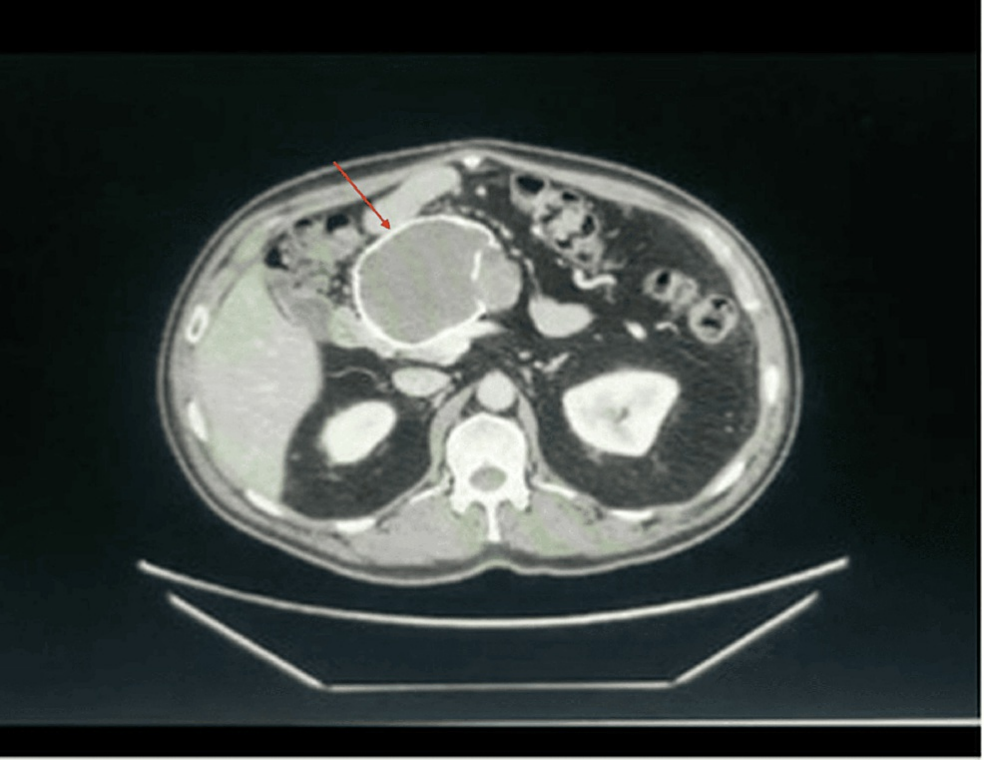
Contrast-enhanced CT scan of the abdomen from 2023. The image reveals a large (9 cm) non-enhancing septated lesion in the head of the pancreas, featuring hyperdense internal material, a thick rim, and coarse calcifications within the septa. Notable atrophy of the pancreatic parenchyma is also observed. There has been no significant change in the size of the lesion compared to the prior exam in 2019 and the initial imaging in 2006.

Fourteen years later, at the age of 55, the patient was seen by a gastroenterologist primarily for persistent episodes of colitis. At this time, the patient also quit alcohol use and developed diabetes mellitus. An abdominal CT scan showed a calcified mass, and a subsequent EUS showed a necrotic, calcified, and vascular mass at the pancreatic head. The FNA biopsy results were inconclusive. A year later, he underwent another EUS/FNA that showed cells positive for beta-catenin and focally positive for CD10, suggesting an SPN. The patient was lost to follow-up and presented four years later, at the age of 59, where an updated CT (Figure 1) continued to show the lesion with no notable change in size but with a slightly hyperdense internal material and a thick rim with septal coarse calcifications.

**Figure 2:**
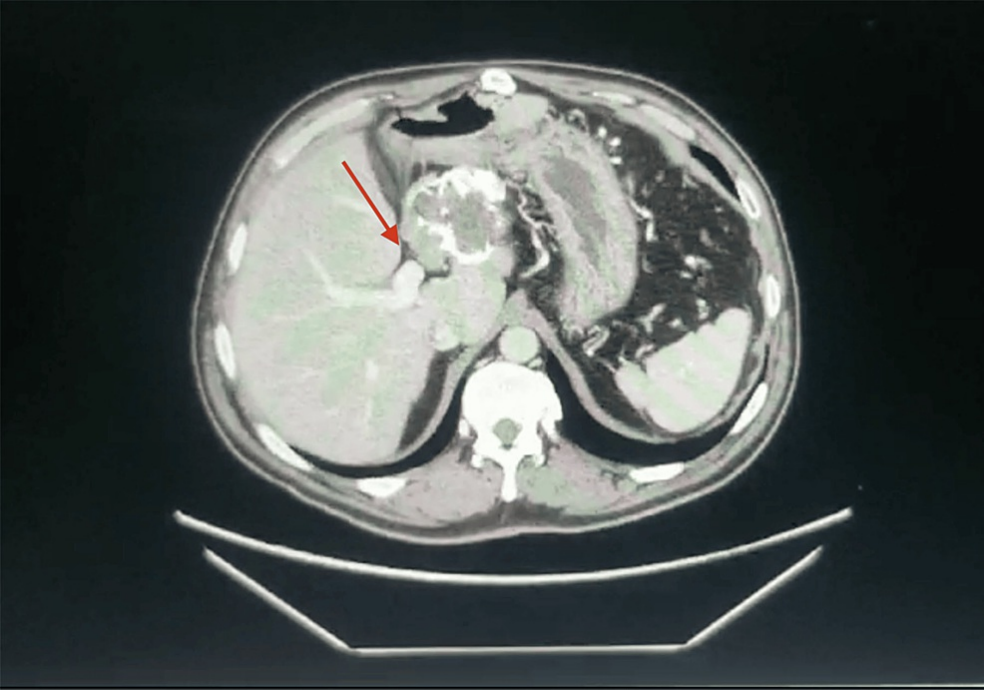
Contrast-enhanced CT scan of the abdomen from 2023. This second view identifies the portal vein in relation to the SPN. SPN, solid pseudopapillary neoplasm

The patient underwent pancreaticoduodenectomy with portal vein resection and reconstruction using a synthetic polyester graft (10 mm Dacron®). Even though the portal vein can generally be dissected off pancreatic SPNs, the portal vein was intimately adherent and not separable from the pancreatic head lesion (Figure 2). This extensive fibrosis is likely related to prior surgical dissection as well as possibly tumor features (fibrosis and osseous metaplasia) discovered on a histologic exam after the resection. Notably, the rest of the pancreatic tissue was atrophic, with no pancreatic tissue left after resecting the uncinate process, and thus, a pancreatojejunostomy was not performed. On postoperative day 1, the patient developed a portal vein thrombosis, promptly underwent a successful portal vein thrombectomy, and was discharged home on postoperative day 11 on a direct oral anticoagulant (Apixaban; Eliquis®).

**Figure 3:**
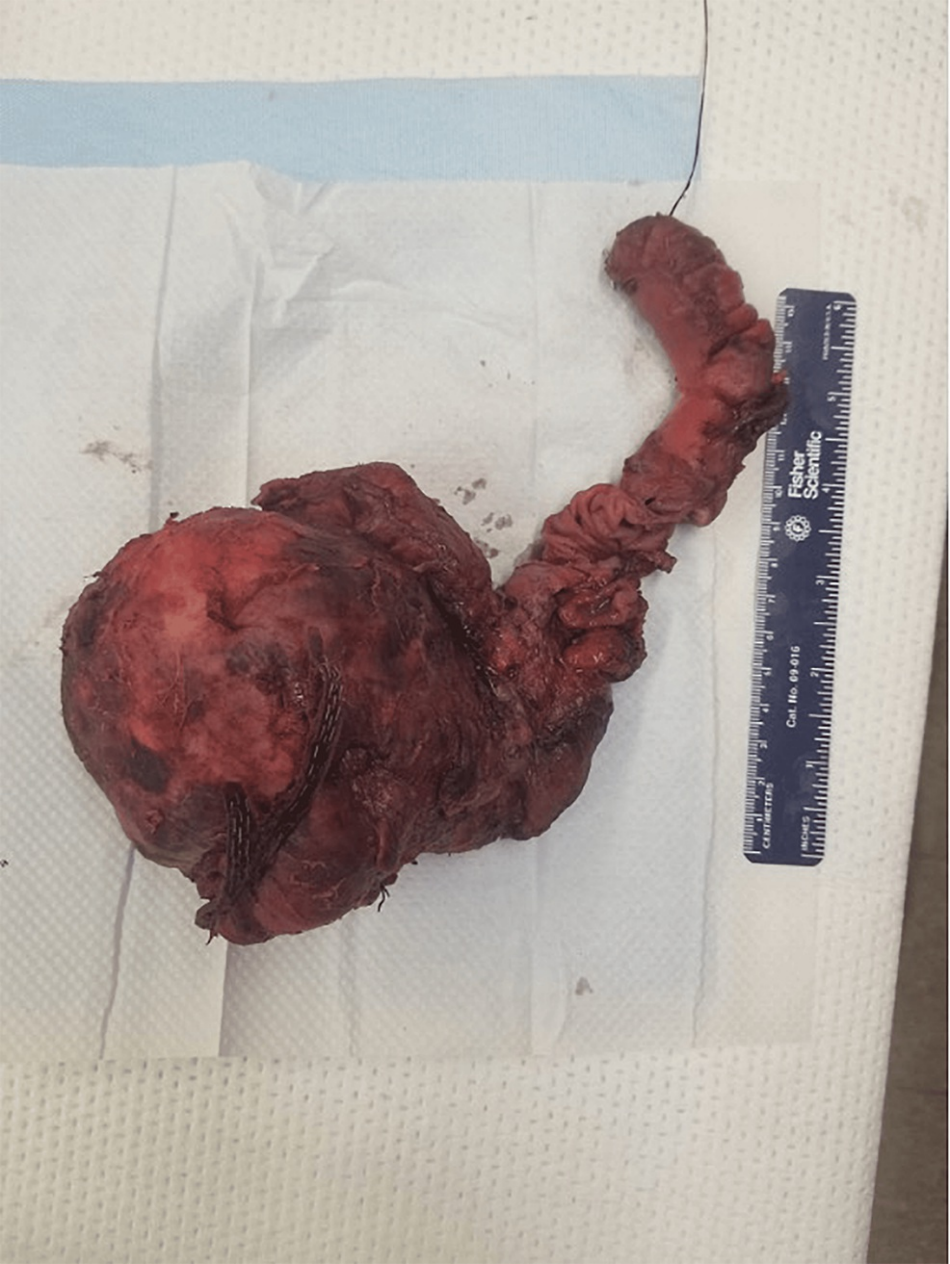
Gross specimen of the pancreaticoduodenectomy. The tumor exhibits a hard, heavily calcified wall.

**Figure 4:**
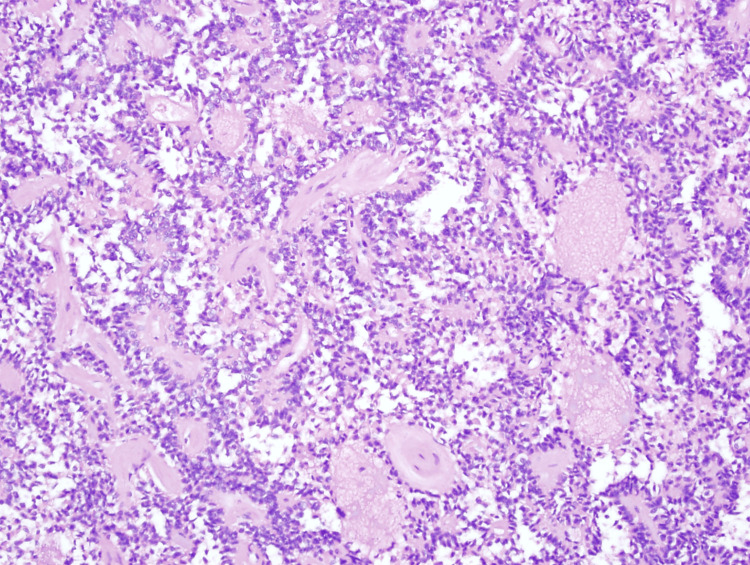
H&E stain of the patient’s SPN of the pancreas at 100x magnification. SPN, solid pseudopapillary neoplasm

Grossly, the tumor was 10 cm in diameter and confined to the pancreas. It appeared cystic, with the cyst wall heavily calcified (Figure 3). Pathological findings confirmed the initial diagnosis of SPN, with the tumor showing a pseudopapillary architectural pattern and tumor cells growing around fibrovascular cores (Figure 4). The cells had mildly elongated and occasionally grooved nuclei of small to intermediate size, with the cytoplasm generally eosinophilic. The wall demonstrated areas of osseous metaplasia. Immunoperoxidase stain results demonstrated positive beta-catenin, CD10, α-1 antitrypsin (AAT), and focally positive pancytokeratin. Additionally, the tumor showed coagulative-type necrosis. All margins were negative for invasive carcinoma; no lymphovascular or perineural invasion was identified, and Ki67 was reported as 1-2%.

## Discussion

The cellular origin of SPNs is unclear but potentially involves ductal, acinar, endocrine, and multipotential stem cells [8]. While SPNs are rare, they are described as prevalent in young women around 25 years of age, with a female predominance [5]. On imaging, SPNs are usually described as well circumscribed, encapsulated, and heterogeneous with hemorrhagic and cystic degeneration and commonly have calcification within and around the periphery of the tumor [9]. The case presented is unique, with the SPN presenting in a middle-aged man and located at the head of the pancreas. Consistent with the literature, SPNs occurring in males tend to present at an older age than their female counterparts.

The case presented with the tumor located at the head of the pancreas, where most cases of SPNs, regardless of gender, are reported to commonly occur within the body and tail [10]. Although the tumor’s location has not been proven to influence the aggressiveness of the disease, it modifies the management with lesions in the head of the pancreas requiring a pancreaticoduodenectomy, while a distal pancreatectomy is considered in tumors of the body or tail [11]. Early referral and management of complex pancreatic tumors by a specialized team could have altered the magnitude and complexity of the surgery, possibly avoiding portal vein resection that was deemed necessary when operating in a previously violated field with scarring. Notably, there was no residual pancreatic tissue, and thus, a pancreaticojejunostomy was not performed. Due to the lack of tissue, there was no need for either a pancreatic drainage procedure or a pancreatic duct occlusion to be performed, and a decision was made to place a drain that was checked for amylase in the postoperative period, confirming no evidence of residual pancreatic tissue. Despite the growing use of pancreatic duct occlusion during a pancreaticoduodenectomy, this was not the case with this patient, and to our knowledge, this is the first case of such an occurrence [12].

SPN evaluation is done preoperatively via EUS/FNA biopsy and confirmed through examination of the resected specimen post-operatively [13-15]. The pathologic, histologic, and immunohistochemical composition is determined by traditionally analyzing FNA samples for amylase, CEA, CA19-9, glucose levels, and oncogene markers (i.e., KRAS, GNAS, VHL, and CTNNB10), with SPN’s staining positive for beta-catenin, CD10, AAT, and variable expression of neuroendocrine markers and cytokeratin [15-18]. Resected specimens demonstrate classic findings, as in the case presented.

In the literature, the rate of malignancy is about 10-15% and is highly associated with tumor size larger than 5 cm, lymphovascular invasion, pancreatic parenchymal invasion, a Ki-67 index greater than 3%, or the presence of metastasis at presentation as described in the WHO malignancy criteria [6-8,19-22]. Both males and females tend to have a low malignancy risk. However, some reports have suggested that male patients may be at higher risk for a more aggressive disease [19]. The risk for recurrence is increased with tumor partial resection [23]. In this case, while the patient is a male with a very large tumor (9 cm), it remained overall stable for over 20 years, and he presented with local disease with no metastasis. Since the entirety of the tumor was resected and had favorable histological features, this patient is considered cured. Thus, overall, the risk of malignancy remains poorly defined, and future research should focus on defining more sensitive markers associated with high-risk and aggressive SPNs.

## Conclusions

SPNs of the pancreas should always be considered in the differential diagnosis when solid cystic lesions are discovered, regardless of age, gender, and location. This case demonstrated that despite a relatively large lesion size presenting in a male patient with extended duration, it still had favorable features reflecting the lack of specific preoperative markers for aggressive tumors, and thus, unless there is an absolute contraindication, it remains to be advised that all SPNs should be completely resected. Managing complex pancreatic pathology is best dealt with by a team handling such cases on a regular basis to provide a curative resection on the first attempt, minimizing the need for a formidable redo.

## Appendices

Appendix A

**Table 1:** Key demographic and clinical features of patients with pancreatic cystic neoplasms IPMN, intraductal papillary mucinous neoplasm; MCN, mucinous cystic neoplasm; SCN, serous cystic neoplasm; SPN, solid pseudopapillary neoplasm Source: Hu et al. (2022) [3]; reproduced with permission from Frontiers

Characteristics	IPMN	MCN	SCN	SPN
Age	50-70s	40-50s	50-60s	20-30s
Gender	Equal	95% female	70% female	90% female
Tumor location	Pancreatic head	Body and tail	Entire pancreas	Body and tail
Imaging features	Multiple mural nodules, pancreatic duct dilation, ductal communication, cyst or cluster of cysts	Large cysts with thick septae, peripheral calcification, and mural nodules	Star-shaped central scar with calcification, microcystic multiple small cyst, sometimes oligocytic	Local capsule interruption, cystic degeneration, calcification and hemorrhage, floating cloud sign
Cyst fluid	Viscous, mucin-rich	Viscous, mucin-rich	Thin	Bloody
Clinical presentation	Jaundice, pancreatitis, malignancy related	Abdominal pain, malignancy related	Abdominal pain, mass effect	Abdominal pain, mass effect
Solitary or multifocal	Solitary/multifocal	Solitary	Solitary	Solitary
CEA	>192-200 ng/ml	>192-200 ng/ml	<5 ng/ml	Unknown
Amylase	High	Low	Low	Low
Molecular markers	KRAS+, GNAS+	KRAS+, GNAS-, CTNNB1-	VHL, VEGF-A>8500pg/mL, VEGF-C>200pg/mL, MUC1, MUC6	CTNNB1, B-Catenin, LEF1, TFE3S, SOX11
Cytology	Columnar cells, +Mucin, variable atypia	Columnar cells, +Mucin, variable atypia	Often acellular or cuboidal cells stain, +glycogen	Branching papillae with myxoid stroma
Malignant probability	Medium or high	Medium	Negligible	Low or medium
